# Supplementary material for: Cancer-associated hypersialylated MUC1 drives the differentiation of human monocytes into macrophages with a pathogenic phenotype
Source: Commun Biol. 2020 Nov 4;3:644. doi: 10.1038/s42003-020-01359-5 (PMC7642421; doi:10.1038/s42003-020-01359-5)
Supplement: Supplementary file 1 — Supplementary Information [file 42003_2020_1359_MOESM1_ESM.pdf]

Cancer-associated hypersialylated MUC1 drives the differentiation of human monocytes into macrophages with a pathogenic phenotype

Richard Beatson[1], Rosalind Graham[1], Fabio Grundland Freile[1], Domenico Cozzetto[2], Shichina Kannambath[3], Ester Pfeifer[1], Natalie Woodman[4], Julie Owen[4], Rosamond Nuamah[3], Ulla Mandel[5], Sarah Pinder[6], Cheryl Gillett[4], Thomas Noll[7], Ihssane Bouybayoune[6], Joyce Taylor-Papadimitriou[1], Joy M. Burchell[1*].

Supplementary figures 1-5 and Supplementary table 1

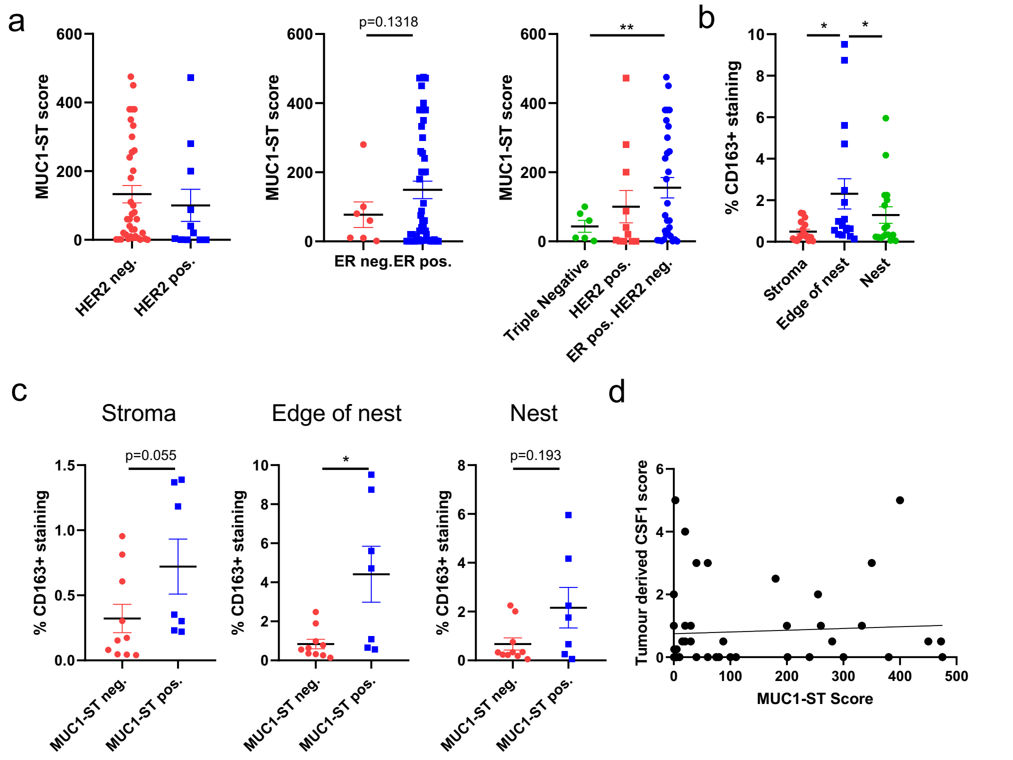

Supplementary Figure 1. Expression of MUC1-ST in breast cancer subtype and of MUC1-ST induced macrophages in tumour areas. **(a)** Pathological subtyping in relation to manual MUC1-ST scoring (n=53). **(b)** Visiopharm automated scoring; % CD163 staining in different regions of tumours (n=17). **(c)** Visiopharm automated scoring; % CD163 staining in MUC1-ST positive (manual score >5) or negative (manual score <5) tumours in the indicated regions (n=17). **(d)** Correlation between manual MUC1-ST score and manual tumour-derived CSF1 score (n=53). Standard error of mean shown. (a-b) *p<0.05, **p<0.01 using unpaired t-test with Welch's correction owing to unequal population variance. (c) *p<0.05 Mann Whitney test (used as populations not Gaussian). Correlations were analysed using linear regression analysis (Pearson's).

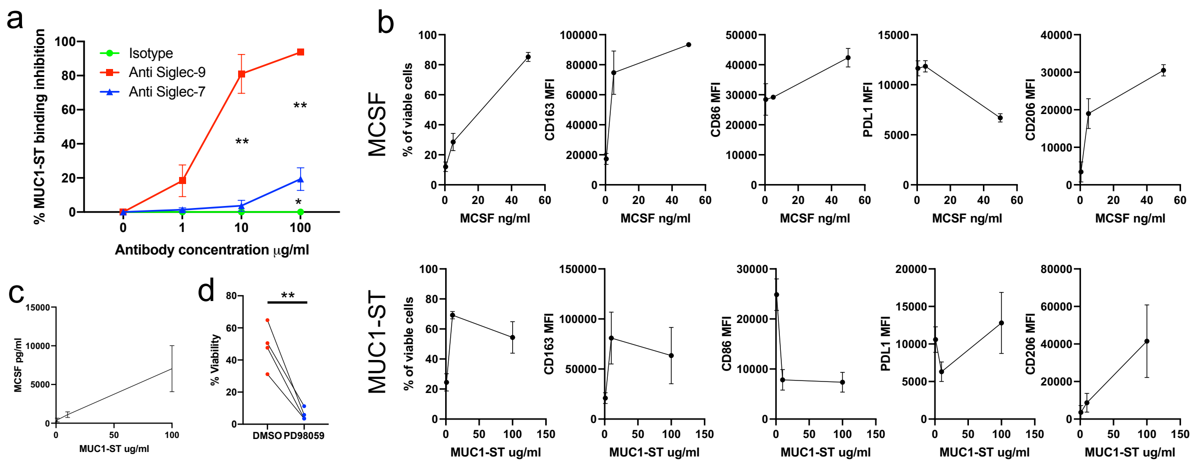

Supplementary Figure 2. Characterisation of MUC1-ST induced monocytes compared to MCSF monocytes and the effect of MEK/ERK inhibitor PD98059. **(a)** % MUC1-ST binding inhibition after pre-incubating monocytes with indicated antibodies at indicated concentrations (n=3). **(b)** The effect of different concentrations of MCSF (top row) and MUC1-ST (bottom row) on monocyte viability and phenotype (n=3). **(c)** The effect of different concentrations of MUC1-ST on MCSF release from monocytes at 48h (n=3). (**d**) Number and viability of monocytes cultured with MUC1-ST for 7 days in serum-free media after pre-incubation with PD98059 or vehicle (n=4). Standard error of mean shown and paired t-test used for statistical analysis. *p<0.05 **p<0.01.

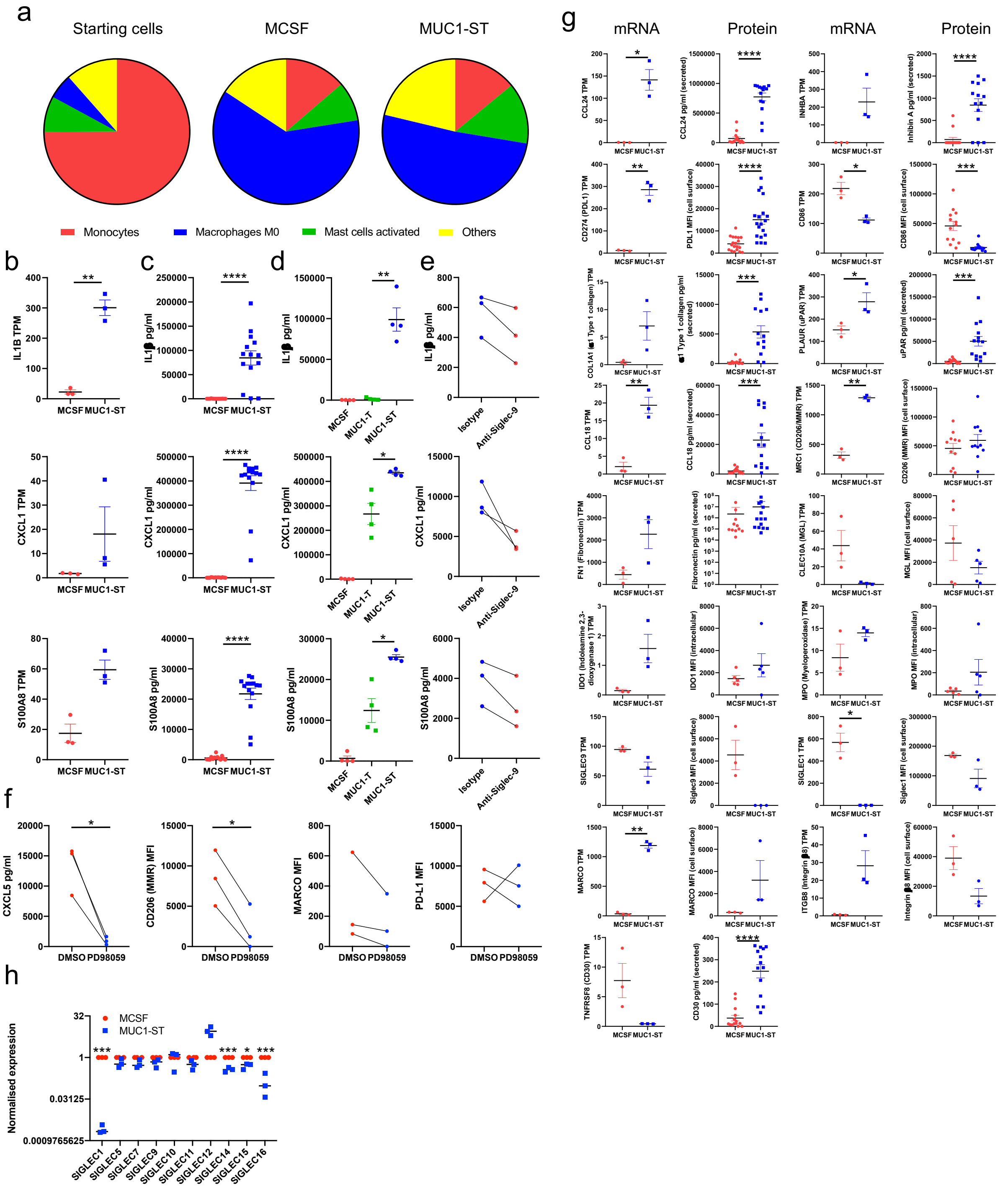

Supplementary Figure 3. Characterisation of MUC1-ST macrophages. **(a)** Determination of immune cell subtype by applying CIBERSORT to starting monocytes (n=2) and monocytes treated with MCSF (n=3) or MUC1-ST (n=3) for 7 days **(b,c,d,e).** Three genes that are differentially expressed between MCSF macrophages and MUC1-ST macrophages **(b)** at the RNA level (n=3), **(c)** and at the protein level (n=14). **(d)** The same 3 proteins assessed in monocytes treated with desialylated MUC1-ST (MUC1-T) (n=4). **(e)** the effect of pre-incubation of monocytes with anti-Siglec-9 or isotype control, prior to MUC1-ST addition, on the production of these 3 proteins (n=3). **(f)** Assessment of an additional 17 proteins whose genes were differentially expressed between MCSF macrophages and MUC1-ST macrophages (n=3-14). **(g)** Levels of CXCL5 in the supernatant and CD206, MARCO and PD-L1 on live macrophages after incubation with PD98059 (a MEK/ERK inhibitor) prior to addition of MUC1-ST and culture for 7 days (n=3). **(h)** Siglec transcript expression levels in monocytes treated with MCSF (n=3) or MUC1-ST (n=3) for 7 days. Standard error of means shown. *p<0.05 **p<0.01 ***p<0.001, ****p<0.0001 using paired t-test. TPM; transcripts per million. MFI. Mean fluorescence intensity

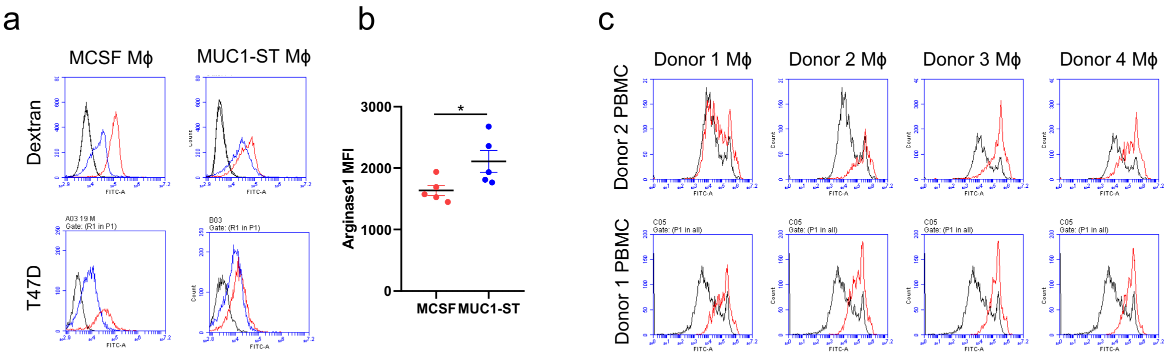

Supplementary Figure 4. Functional characterisation of MUÇ-ST induced macrophages. **(a)** representative histograms showing dextran-FITC uptake (top; blue; 4C. red; 37C) and the uptake of labelled T47D tumour cells (bottom; blue; 4C. red; 37C) by MCSF macrophages and MUC1-ST macrophages after 4h incubation, against macrophages alone (black). **(b)** intracellular staining of arginase in MCSF macrophages and MUC1-ST macrophages (n=5). **(c)** representative histograms showing the effect of media alone (black) or MUC1-ST (red) macrophage supernatant on the proliferation of CD3 stimulated PBMCs after 4 days. Standard error of mean shown, *p<0.05 using paired t-test.

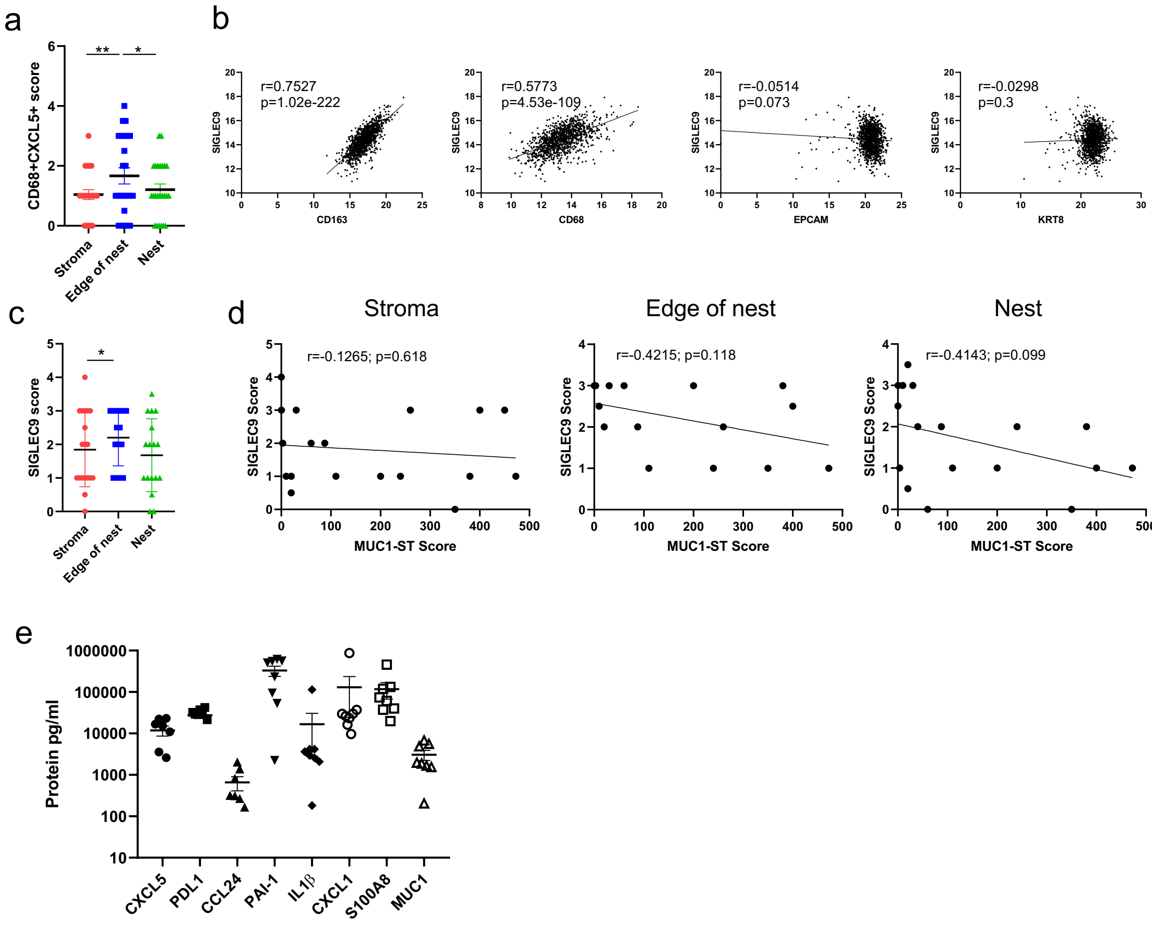

Supplementary Figure 5. Location of macrophages and Siglec-9 expression within the tumour. **(a)** Manual scoring of CD68+CXCL5+ cells in different indicated regions of the tumour (n=24). **(b)** TCGA data showing the correlation between SIGLEC9 and CD163, CD68, and EPCAM and KRT8 expression levels in breast cancers (n=1217). **(c)** Manual scoring of SIGLEC9+ cells in different indicated regions of the tumour (stroma n=18, edge n=15, nest n=17). **(d)** SIGLEC9 manual scores in different indicated regions of the tumour measured against MUC1-ST manual scoring (stroma n=18, edge n=15, nest n=17. **(e)** Measurement of indicated factors in the interstitial fluid of fresh breast cancers (n=8). Standard error of the mean shown and paired t-test used *p<0.05, **p<0.01. Correlations were analysed using linear regression analysis (Pearson's).

Antigen	Clone	Host species	Subtype	Conjugate	Company	Catalogue number
Arginase1	A1exF5	Rat	IgG2a	PerCP	ebioscience	46-3697-81
CD11b	ICRF44	mouse	IgG1	APC	Biolegend	301309
CD14	M5E2	mouse	IgG2a	FITC	Biolegend	301804
CD15	HI98	mouse	IgM	PE	Biolegend	301906
CD16	B73.1	mouse	IgG1	FITC	Biolegend	360715
CD163	MRQ-26	mouse	IgG1	Unconjugated	Roche	760-4437
CD163	GHI-61	mouse	IgG1	FITC	Biolegend	333618
CD206	15-2	mouse	IgG1	PerCP	Biolegend	321122
CD3	OKT3	mouse	IgG2a	Unconjugated	Biolegend	317325
CD30	BY88	mouse	IgG1	PE	Biolegend	333906
CD38	HIT2	mouse	IgG1	PE	Biolegend	303505
CD66b	G10F5	mouse	IgM	PerCP	Biolegend	305108
CD68	KP1	mouse	IgG1	Unconjugated	Roche	790-2931
CD68	KP1	mouse	IgG1	Unconjugated	ebioscience	14-0688-82
CD86	IT2.2	mouse	IgG2b	PE	Biolegend	305406
CXCL5	Polyclonal	goat	IgG	Unconjugated	Biotechne	AF254
Goat IgG	Polyclonal	donkey	IgG	NL557	Biotechne	NL001
IDO1	eyedio	mouse	IgG1	PE	ebioscience	12-9477-41
Integrin 8	416922	mouse	IgG2b	Unconjugated	Biotechne	MAB4775
MARCO	PLK-1	mouse	IgG3	PE	ebioscience	12-5447-41
MCSF	A16067H	mouse	IgG2b	Unconjugated	Biolegend	699203
MGL	H037G3	mouse	IgG2a	PE	Biolegend	354704
Mouse Ig	Polyclonal	rabbit	Ig	HRP	DAKO	P0260
Mouse Ig	Polyclonal	goat	Ig	HRP	DAKO	P0447
Mouse Ig	Polyclonal	rabbit	Ig	FITC	DAKO	F0261
Mouse IgG	Polyclonal	donkey	IgG	Alexa Fluor 488	ThermoFisher	A-21202
MPO	MPO455-8E6	mouse	IgG1	FITC	ebioscience	11-1299-41
MUC1-T	1B9	mouse	IgG1	Unconjugated	NA	NA
NA	MOPC-21	mouse	IgG1	PerCP	Biolegend	400148
NA	MOPC-21	mouse	IgG1	FITC	Biolegend	400108
NA	MOPC-21	mouse	IgG1	Unconjugated	Biolegend	400124
NA	MOPC-21	mouse	IgG1	PE	Biolegend	400112
NA	MOPC-21	mouse	IgG1	APC	Biolegend	400120
NA	MOPC-173	mouse	IgG2a	FITC	Biolegend	400210
NA	MG2a-53	mouse	IgG2a	Unconjugated	Biolegend	401504
NA	MOPC-173	mouse	IgG2a	PerCP	Biolegend	400250
NA	MOPC-173	mouse	IgG2a	PE	Biolegend	400214
NA	RTK2758	rat	IgG2a	PerCP	Biolegend	400529
NA	MPC-11	mouse	IgG2b	Unconjugated	Biolegend	400347
NA	MPC-11	mouse	IgG2b	PerCP	Biolegend	400336
NA	20116	mouse	IgG2b	Unconjugated	Biotechne	MAB004
NA	MPC-11	mouse	IgG2b	PE	Biolegend	400314
NA	MG3-35	mouse	IgG3	PE	Biolegend	401319
NA	MM-30	mouse	IgM	PerCP	Biolegend	401623
NA	MM-30	mouse	IgM	PE	Biolegend	401611
PDL1	29E.2A3	mouse	IgG2b	PerCP	Biolegend	329738
Siglec 1	HSn 7D2	mouse	IgG1	Unconjugated	Abcam	Ab18619
Siglec 9	191240	mouse	IgG2a	Unconjugated	Biotechne	MAB1139
Siglec 9	191240	mouse	IgG2a	FITC	Biotechne	FAB1139F
Tissue Factor	NY2	mouse	IgG1	APC	Biolegend	365205

Supplementary Table 1: Antibodies used in the study
