# Supplementary material for: Cancer-associated hypersialylated MUC1 drives the differentiation of human monocytes into macrophages with a pathogenic phenotype
Source: Commun Biol. 2020 Nov 4;3:644. doi: 10.1038/s42003-020-01359-5 (PMC7642421; doi:10.1038/s42003-020-01359-5)
Supplement: Supplementary file 3 — Description of Additional Supplementary Files [file 42003_2020_1359_MOESM3_ESM.pdf]

Description of Additional Supplementary Files File

Name: Supplementary Data 1

Description: RNAseq data

Name: Supplementary Data 2

Description: Data for figures in main text
