# Supplementary material for: Cancer-associated hypersialylated MUC1 drives the differentiation of human monocytes into macrophages with a pathogenic phenotype
Source: Commun Biol. 2020 Nov 4;3:644. doi: 10.1038/s42003-020-01359-5 (PMC7642421; doi:10.1038/s42003-020-01359-5)
Supplement: Supplementary file 6 — Reporting Summary [file 42003_2020_1359_MOESM6_ESM.pdf]

nature research

Corresponding author(s): Joy Burchell

Last updated by author(s): 15th September 2020

Reporting Summary

Nature Research wishes to improve the reproducibility of the work that we publish. This form provides structure for consistency and transparency in reporting. For further information on Nature Research policies, see Authors & Referees and the Editorial Policy Checklist.

Statistics

For all statistical analyses, confirm that the following items are present in the figure legend, table legend, main text, or Methods section.

n/a	Confirmed	
☐	☒	The exact sample size (n) for each experimental group/condition, given as a discrete number and unit of measurement
☐	☒	A statement on whether measurements were taken from distinct samples or whether the same sample was measured repeatedly
☐	☒	The statistical test(s) used AND whether they are one- or two-sided<br>*Only common tests should be described solely by name; describe more complex techniques in the Methods section.*
☐	☒	A description of all covariates tested
☒	☐	A description of any assumptions or corrections, such as tests of normality and adjustment for multiple comparisons
☐	☒	A full description of the statistical parameters including central tendency (e.g. means) or other basic estimates (e.g. regression coefficient) AND variation (e.g. standard deviation) or associated estimates of uncertainty (e.g. confidence intervals)
☒	☐	For null hypothesis testing, the test statistic (e.g. F, t, r) with confidence intervals, effect sizes, degrees of freedom and P value noted<br>*Give P values as exact values whenever suitable.*
☒	☐	For Bayesian analysis, information on the choice of priors and Markov chain Monte Carlo settings
☒	☐	For hierarchical and complex designs, identification of the appropriate level for tests and full reporting of outcomes
☐	☒	Estimates of effect sizes (e.g. Cohen's d, Pearson's r), indicating how they were calculated

Our web collection on statistics for biologists contains articles on many of the points above.

Software and code

Policy information about availability of computer code

Data collection	*Provide a description of all commercial, open source and custom code used to collect the data in this study, specifying the version used OR state that no software was used.*
Data analysis	The CIBERSORT R source code and the LM22 signature matrix file, which defines 22 immune cell types based on the expression levels of 547 genes, were downloaded from https://cibersort.stanford.edu/.

For manuscripts utilizing custom algorithms or software that are central to the research but not yet described in published literature, software must be made available to editors/reviewers. We strongly encourage code deposition in a community repository (e.g. GitHub). See the Nature Research guidelines for submitting code & software for further information.

Data

Policy information about availability of data

All manuscripts must include a data availability statement. This statement should provide the following information, where applicable:

- Accession codes, unique identifiers, or web links for publicly available datasets
- A list of figures that have associated raw data
- A description of any restrictions on data availability

The RNAseq data that support the findings will be available in GEO at (TBD) following a 6 month embargo from the date of publication to allow for potential commercialization of research findings. All other data that support the findings of this study are available from the corresponding author, [JB], upon reasonable request.

Field-specific reporting

Please select the one below that is the best fit for your research. If you are not sure, read the appropriate sections before making your selection.

☒ Life sciences ☐ Behavioural & social sciences ☐ Ecological, evolutionary & environmental sciences

For a reference copy of the document with all sections, see nature.com/documents/nr-reporting-summary-flat.pdf

Life sciences study design

All studies must disclose on these points even when the disclosure is negative.

Sample size	Multiple sample sizes were used in this publication.<br>60 breast cancer cases (7 lost owing to poor tissue quality, hence n=53) - sufficient for linear regression analysis<br>3 matched donors RNAseq - with good depth of read, sufficient to observe statistically significant differences<br>3-14 donors for cellular work - minimum 3 to enable statistical analysis.<br>15-18 for BASEscope of Siglec9 - loss of adequate tissue definition owing to over-digestion for some cases - sufficient for trends to be observed.<br>8 for interstitial fluid - availability of samples - sufficient for statistical analysis<br>1217 for TCGA analysis - entire dataset included - sufficient for analysis<br>24 for CD68 and CXCL5 analysis - resourcing - sufficient for trends to be observed.<br>17 for Visiopharm - resourcing - sufficient for analysis.
Data exclusions	One data point was excluded from figure 2b - reason, it was an extreme outlier as determined using Grubb's test.
Replication	All experiments were replicated at least once, excluding the RNAseq, some replicated >10 times. All replicates are included in the data presented in figures
Randomization	No need for randomisation as all donors were unique, none repeated, and we were blinded from any personal information.
Blinding	Blinded from personal information with respect to breast cancer cases and donor blood. Blinded to pathological and clinical data of breast cancer samples until experiments completed.

Reporting for specific materials, systems and methods

We require information from authors about some types of materials, experimental systems and methods used in many studies. Here, indicate whether each material, system or method listed is relevant to your study. If you are not sure if a list item applies to your research, read the appropriate section before selecting a response.

Materials & experimental systems

n/a	Involved in the study	
☐	☒	Antibodies
☐	☒	Eukaryotic cell lines
☒	☐	Palaeontology
☒	☐	Animals and other organisms
☒	☐	Human research participants
☒	☐	Clinical data

Methods

n/a	Involved in the study	
☒	☐	ChIP-seq
☐	☒	Flow cytometry
☒	☐	MRI-based neuroimaging

Antibodies

Antibodies used	Antigen Clone Host species Subtype Conjugate Company Catalogue number<br>Arginase1 A1exF5 Rat IgG2a PerCP ebioscience 46-3697-81<br>CD11b ICRF44 mouse IgG1 APC Biolegend 301309<br>CD14 M5E2 mouse IgG2a FITC Biolegend 301804<br>CD15 HI98 mouse IgM PE Biolegend 301906<br>CD16 B73.1 mouse IgG1 FITC Biolegend 360715<br>CD163 MRQ-26 mouse IgG1 Unconjugated Roche 760-4437<br>CD163 GHI-61 mouse IgG1 FITC Biolegend 333618<br>CD206 15-2 mouse IgG1 PerCP Biolegend 321122<br>CD3 OKT3 mouse IgG2a Unconjugated Biolegend 317325<br>CD30 BY88 mouse IgG1 PE Biolegend 333906<br>CD38 HIT2 mouse IgG1 PE Biolegend 303505<br>CD66b G10F5 mouse IgM PerCP Biolegend 305108

CD68 KP1 mouse IgG1 Unconjugated Roche 790-2931
CD68 KP1 mouse IgG1 Unconjugated ebioscience 14-0688-82
CD86 IT2.2 mouse IgG2b PE Biolegend 305406
CXCL5 Polyclonal goat IgG Unconjugated Biotechne AF254
Goat IgG Polyclonal donkey IgG NL557 Biotechne NL001
IDO1 eyedio mouse IgG1 PE ebioscience 12-9477-41
Integrin 8 416922 mouse IgG2b Unconjugated Biotechne MAB4775
MARCO PLK-1 mouse IgG3 PE ebioscience 12-5447-41
MCSF A16067H mouse IgG2b Unconjugated Biolegend 699203
MGL H037G3 mouse IgG2a PE Biolegend 354704
Mouse Ig Polyclonal rabbit Ig HRP DAKO P0260
Mouse Ig Polyclonal goat Ig HRP DAKO P0447
Mouse Ig Polyclonal rabbit Ig FITC DAKO F0261
Mouse IgG Polyclonal donkey IgG Alexa Fluor 488 ThermoFisher A-21202
MPO MPO455-8E6 mouse IgG1 FITC ebioscience 11-1299-41
MUC1-T 1B9 mouse IgG1 Unconjugated NA NA
NA MOPC-21 mouse IgG1 PerCP Biolegend 400148
NA MOPC-21 mouse IgG1 FITC Biolegend 400108
NA MOPC-21 mouse IgG1 Unconjugated Biolegend 400124
NA MOPC-21 mouse IgG1 PE Biolegend 400112
NA MOPC-21 mouse IgG1 APC Biolegend 400120
NA MOPC-173 mouse IgG2a FITC Biolegend 400210
NA MG2a-53 mouse IgG2a Unconjugated Biolegend 401504
NA MOPC-173 mouse IgG2a PerCP Biolegend 400250
NA MOPC-173 mouse IgG2a PE Biolegend 400214
NA RTK2758 rat IgG2a PerCP Biolegend 400529
NA MPC-11 mouse IgG2b Unconjugated Biolegend 400347
NA MPC-11 mouse IgG2b PerCP Biolegend 400336
NA 20116 mouse IgG2b Unconjugated Biotechne MAB004
NA MPC-11 mouse IgG2b PE Biolegend 400314
NA MG3-35 mouse IgG3 PE Biolegend 401319
NA MM-30 mouse IgM PerCP Biolegend 401623
NA MM-30 mouse IgM PE Biolegend 401611
PDL1 29E.2A3 mouse IgG2b PerCP Biolegend 329738
Siglec 1 HSn 7D2 mouse IgG1 Unconjugated Abcam Ab18619
Siglec 9 191240 mouse IgG2a Unconjugated Biotechne MAB1139
Siglec 9 191240 mouse IgG2a FITC Biotechne FAB1139F
Tissue Factor NY2 mouse IgG1 APC Biolegend 365205

Validation

All validated for appropriate use by the company listed, or by ourselves and our collaborators (MUC1T; 1B9)

Eukaryotic cell lines

Policy information about cell lines

Cell line source(s)

T47D were obtained from their originator; Dr Keydar. MCF7 cells from ATCC

Authentication

MCF-7 cells were authenticated by LGC Standards in February 2011 using the short-tandem repeat profile of 16 loci. These contain the 9 loci used by the American Type Culture Collection and both lines exactly matched these profiles.
T47D have not been authenticated but have never been keep in culture for longer than 6-8weeks.

Mycoplasma contamination

All lines were negative for mycoplasma contamination

Commonly misidentified lines
(See ICLAC register)

Name any commonly misidentified cell lines used in the study and provide a rationale for their use.

Flow Cytometry

Plots

Confirm that:

☐ The axis labels state the marker and fluorochrome used (e.g. CD4-FITC).

☐ The axis scales are clearly visible. Include numbers along axes only for bottom left plot of group (a 'group' is an analysis of identical markers).

☐ All plots are contour plots with outliers or pseudocolor plots.

☐ A numerical value for number of cells or percentage (with statistics) is provided.

Methodology

Sample preparation

1x105 cells were stained with a live/dead dye (ThermoFisher; L23102) in PBS for 10 minutes on ice in the dark, before being

Sample preparation	washed twice in FACS buffer (0.5% BSA [Sigma; 05482] in PBS + 2mM EDTA). Cells were then Fc blocked with Trustain (Biolegend; 17422302) in FACS buffer for 10 minutes on ice in the dark. Cells were washed and then stained using a variety of antibodies +/- secondary reagents described above, on ice for 30 minutes in the dark (if secondaries were used, cells were washed in FACS buffer before being further incubated on ice with secondary for 30 minutes). Cells were washed and either read immediately or fixed using 1% PFA in FACS buffer and read within 3 days. Intracellular flow cytometry was carried out using the intracellular fixation and permeabilization kit (ebioscience; 88-8824-00) according to manufacturer's instructions.
Instrument	BD Accuri C6 Plus
Software	BD Accuri C6 Plus software
Cell population abundance	Purity was >95% for starting monocyte population as determined by CD14. This was maintained for the macrophage work. For MLR, there was relative loss (from starting ratio) of PBMC population as shown in the viability data in 5h
Gating strategy	All cells were gated as follows a) FSC and SSC to exclude cellular debris (whilst also adjusting threshold) b) live/dead (only live cells carried forward) c) SSC-A vs SSC-H – only singlets carried forward. All MFIs were corrected against an appropriate isotype control. The isotype control determined if cells were considered positive.

☐ Tick this box to confirm that a figure exemplifying the gating strategy is provided in the Supplementary Information.
